# ONC201/TIC10 Is Empowered by 2-Deoxyglucose and Causes Metabolic Reprogramming in Medulloblastoma Cells *in Vitro* Independent of C-Myc Expression

**DOI:** 10.3389/fcell.2021.734699

**Published:** 2021-11-26

**Authors:** Annika Dwucet, Maximilian Pruss, Qiyu Cao, Mine Tanriover, Varun V. Prabhu, Joshua E. Allen, Aurelia Peraud, Mike-Andrew Westhoff, Markus D. Siegelin, Christian Rainer Wirtz, Georg Karpel-Massler

**Affiliations:** ^1^ Department of Neurosurgery, Ulm University Medical Center, Ulm, Germany; ^2^ Chimerix Inc., Durham, NC, United States; ^3^ Department of Pediatric and Adolescent Medicine, Ulm University Medical Center, Ulm, Germany; ^4^ Department of Pathology and Cell Biology, Columbia University Medical Center, New York, NY, United States

**Keywords:** medulloblastoma, ONC201/TIC10, 2-Deoxyglucose, seahorse, metabolism

## Abstract

The purpose of this study was to examine whether the imipridone ONC201/TIC10 affects the metabolic and proliferative activity of medulloblastoma cells *in vitro*. Preclinical drug testing including extracellular flux analyses (agilent seahorse), MTT assays and Western blot analyses were performed in high and low c-myc-expressing medulloblastoma cells. Our data show that treatment with the imipridone ONC201/TIC10 leads to a significant inihibitory effect on the cellular viability of different medulloblastoma cells independent of c-myc expression. This effect is enhanced by glucose starvation. While ONC201/TIC10 decreases the oxidative consumption rates in D458 (c-myc high) and DAOY (c-myc low) cells extracellular acidification rates experienced an increase in D458 and a decrease in DAOY cells. Combined treatment with ONC201/TIC10 and the glycolysis inhibitor 2-Deoxyglucose led to a synergistic inhibitory effect on the cellular viability of medulloblastoma cells including spheroid models. In conclusion, our data suggest that ONC201/TIC10 has a profound anti-proliferative activity against medulloblastoma cells independent of c-myc expression. Metabolic targeting of medulloblastoma cells by ONC201/TIC10 can be significantly enhanced by an additional treatment with the glycolysis inhibitor 2-Deoxyglucose. Further investigations are warranted.

## Introduction

Medulloblastoma represents a heterogenous tumor entity that constitutes more than 60% of embryonal brain tumors in children (Khanna et al., 2017; Northcott et al., 2019). This disease is mostly localized in the cerebellum and the majority of medulloblastomas occur in pediatric patients with a median age of 9 years (Roberts et al., 1991; Orr, 2020). Despite the fact that the majority of these tumors arise in children, adults are also rarely affected by this disease (Giordana et al., 1999). Based on distinct molecular characteristics, medulloblastomas can be divided into the WNT, sonic hedgehog (SHH), group 3 and group 4 subgroups (Taylor et al., 2011). While current first-line therapy is associated with a 5-year overall survival greater than 70% in standard-risk patients, high-risk patients face a significantly inferior clinical course (Oyharcabal-Bourden et al., 2005; Gajjar et al., 2006; Packer et al., 2006; Cavalli et al., 2017; Khanna et al., 2017). In addition, first-line therapy is associated with an important side-effect burden including neurocognitive and endocrinological deficits as well as a strong psychosocial impairment. Notably, patients with group 3 myc-amplified or myc-overexpressing tumors were identified to be of high-risk facing a worse clinical outcome (Cavalli et al., 2017; Northcott et al., 2019). As a consequence, novel strategies need to be developed that are taking in account the characteristics of the individual disease in order to increase the therapeutic efficacy and to lower the toxicity.

Alterations with respect to the metabolic activity of cancer cells have already been described in the 1920s (Warburg et al., 1927). Otto Warburg was the first to report that cancer cells preferentially metabolize glucose through glycolysis instead of oxidative phosphorylation despite an abundant presence of oxygen. This metabolic feature is tailored to cope with a great need in biomass in order to maintain a high proliferative activity as typically seen in malignancies (Venneti and Thompson, 2017). Nowadays, it is known that the specific features of the tumor cell metabolism are much more complex and exceed by far the sole phenomenon of aerobic glycolysis (Pavlova and Thompson, 2016). The facts that 1) the cell metabolism represents a center node to maintain the cellular function and 2) cancer cell-specific metabolic features offer potential vulnerabilities support efforts to target the tumor cell metabolism to develop therapeutic strategies against cancer.

ONC201/TIC10, belongs to a class of substances termed imipridones and was discovered to have an anti-cancer activity through a drug screen searching for compounds that induce Tumor Necrosis Factor-related apoptosis-inducing ligand (TRAIL) (Allen et al., 2013). One of the main mechanisms of action of ONC201/TIC10 was discovered to rely on the hyperactivation of the mitochondrial caseinolytic protease P (CIpP) (Ishizawa et al., 2019). In turn, ONC201/TIC10-mediated activation of CIpP was shown to induce a decreased expression of respiratory chain proteins and subsequently, an impaired oxidative phosphorylation. Notably, recent preclinical studies reported that ONC201/TIC10 offers a strong antineoplastic activity against pediatric tumor cells such as diffuse intrinsic pontine glioma or MYCN-amplified neuroblastoma cells (El-Soussi et al., 2021; Zhang et al., 2021). Moreover, high expression of c-myc was shown to be associated with an improved response towards ONC201/TIC10 in glioblastoma models (Ishida et al., 2018). We therefore formed the hypothesis that this forementioned observation may prove favorable to patients with group 3 medulloblastomas in which myc amplification relates to a worse prognosis. From a translational perspective, ONC201/TIC10 has been clinically applied and is currently tested in trials including children with gliomas (NCT02525692, NCT03416530) (Arrillaga-Romany et al., 2017; Stein et al., 2017; Arrillaga et al., 2018; Karpel-Massler and Siegelin, 2018; Chi et al., 2019).

In this study, we provide evidence that ONC201/TIC10 has a strong antiproliferative activity on medulloblastoma cells with IC_50_-values in the lower micromolar range. We also show that this effect is enhanced by glucose deprivation and that its anti-proliferative activity seemed not to be overly affected by c-myc expression. At baseline, extracellular flux analyses showed a consistent down-regulation of oxygen consumption rates following treatment with ONC201/TIC10 among DAOY and D458 cells. In contrast, the response in glycolytic rates varied among the two cell lines tested. Notably, additional treatment with 2-Deoxyglucose led to a synergistic anti-proliferative activity.

## Materials and Methods

### Reagents

ONC201/TIC10 was kindly provided by Oncoceutics, Inc. (Philadelphia, PA, United States). 2-Deoxyglucose was purchased from Sigma Aldrich (St. Louis, MO, United States). A 10 mM stock solution was prepared for ONC201/TIC10 with dimethylsulfoxide (DMSO). For 2-Deoxyglucose a 500 mM stock solution was prepared with sterile water. All stock solutions were stored at −20°C. For all experiments, final concentrations of DMSO were below 0.1% (v/v).

### Cell Cultures and Growth Conditions

D425, D458, and DAOY human medulloblastoma cells were obtained from the American Type Culture Collection (Manassas, VA, United States). HD-MB03 cells were purchased from the German Collection of Microorganisms and Cell Cultures (DSMZ, Braunschweig, Germany). The identities of the medulloblastoma cell lines were confirmed by the source of purchase. MB-PC322 cells were cultivated from tumor tissue obtained from a patient that was operated on at our hospital. The procedure was approved by the ethics committee of the University of Ulm (No.162/10) and consent was granted by next of kin. The initial stocks were expanded, frozen and stored in liquid nitrogen. Fresh aliquots were thawed every 6 weeks. DAOY cells were cultured in Minimum Essential Medium (MEM, Gibco, Grand Island, NY, United States) supplemented with 20% FBS (Gibco, Grand Island, NY, United States), 1% Penicillin/Streptomycin (Gibco, Grand Island, NY, United States), 1% l-glutamine (Gibco, Grand Island, NY, United States), 1% MEM non-essential amino acids (Gibco, Grand Island, NY, United States), 1% sodium pyruvate (Gibco, Grand Island, NY, United States) and 25 mM HEPES (Bioand Sell, Feucht, Germany). D425 and D458 cells were cultured in Improved MEM Zinc Option (IMEMZO, Gibco, Grand Island, NY, United States) supplemented with 20% FBS (Gibco, Grand Island, NY, United States), 1% Penicilline/Streptomycine (Gibco, Grand Island, NY, United States), 1% MEM non-essential amino acids (Gibco, Grand Island, NY, United States) and 25 mM HEPES (Biochrom, Feucht, Germany). HD-MB03 cells were cultured in Roswell Park Memorial Institute Medium (RPMI, Gibco, Grand Island, NY, United States) supplemented with 10% FBS (Gibco, Grand Island, NY, United States) and 1% Penicilline/Streptomycine (Gibco, Grand Island, NY, United States). MB-PC322 cells were cultured in Dulbecco’s Modified Eagle’s Medium (DMEM, Gibco, Grand Island, NY, United States) supplemented with 10% FBS (Gibco, Grand Island, NY, United States) and 1% Penicillin/Streptomycin (Gibco, Grand Island, NY, United States). All cells were cultivated humidified at 37°C and 5% CO_2_.

### Cell Viability Assays

In order to examine cellular proliferation, 3-(4, 5-dimethylthiazol-2-yl)-2, 5-diphenyltetrazolium bromide (MTT) assays were performed as previously described (Karpel-Massler et al., 2015; Pruss et al., 2020).

### Transfections of siRNAs

For siRNA transfections, lipofectamine 3000 (Invitrogen, Carlsbad, CA, United States) was used according to the manufacturer’s instructions. Briefly, 5000 cells/well were seeded in 96 well plates. After 24 h, the siRNA-lipid complex was added to the cells followed by an incubation for 24 h. Then, treatments were performed for 72 h prior to analysis of the cellular viability by MTT assays or protein expression by Western blot. siRNA targeting c-myc was purchased from CST (#6552, c-myc siRNA II, SignalSilence^®^; Cell Signaling Technology, Danvers, MA, United States). Non-targeting siRNA was obtained from Dharmacon (D-001810-03-05, ON-TARGETplus; Lafayette, CO, United States).

### Western Blot Analysis

Specific protein expression in cell lines was determined by Western blot analysis as described before (Hlavac et al., 2019; Pruss et al., 2020) using the following primary antibodies: Total OXPHOS human WB antibody cocktail (1:1,000, #ab110411, Abcam, Cambridge, U.K.), c-myc (1:1,000, #18583, clone E5Q6W; Cell Signaling Technology, Danvers, MA, United States), c-myc/n-myc (1:1,000, #13987, clone D3N8F; Cell Signaling Technology, Danvers, MA, United States) and β-actin (1:2,000, clone AC15; Sigma Aldrich, St. Louis, MO). Secondary HRP-linked antibodies were purchased from Cell Signaling Technology (#7076S, #7074S).

### Extracellular Flux Analysis

1 × 10^4^ cells were seeded on XF96 V3 PS cell culture microplates (Agilent Technologies Inc., Wilmington, DE, United States). After 24 h cells subjected to the indicated treatments for 24 h followed by washes with XF assay medium containing 5 mM glucose (pH adjusted to 7.5). Afterwards the mito stress test kit (Agilent Technologies Inc., Wilmington, DE, United States) was used as described by the manufacturer applying serial injections of oligomycin at a final concentration of 2 μM, FCCP at a final concentration of 2 µM and rotenone/antimycin A at a final concentration of 0.5 µM. All analyses were performed on an Agilent Seahorse XFe96 analyzer.

### Spheroid Assay

Spheroids were established to assess the effects of ONC201/2-Deoxyglucose in a 3-dimensional setting. In 96-well plates, 0.35 × 10^5^ cells/well were resuspended in 20 µL of a mixture of 80% Matrigel and 20% DMEM prior to incubation for 1 h at 37°C. Afterwards, the cell/Matrigel matrix was gently transferred to 12-well plates containing DMEM (10% FBS). Then, spheroids were allowed to grow for 7 days prior to changing the medium to DMEM containing 1.5% FBS and starting treatments. For quantification, CellTiter-Glo^®^ assays were performed. To this purpose, spheroids suspended in 100 µL of medium were transferred to opaque-walled 96-well plates prior to adding 100 µL of the CellTiter-Glo^®^ solution followed by incubation for 10 min at RT and measurement of luminescence.

### Statistical Analysis

Statistical significance was assessed by one-way ANOVA followed by Newman-Keuls post hoc analysis using GraphPad Prism version 5.04 (La Jolla, CA). A *p* ≤ 0.05 was considered statistically significant. Combination indices and isobolograms were calculated using the CompuSyn software (ComboSyn, Inc., Paramus, NJ) as described before (Karpel-Massler et al., 2017). For BLISS analysis, the expected total response was calculated as fractional response to drug A (Fa) + fractional response to drug B (Fb)—Fa x Fb. A ratio of the actual total response and the expected total response of 0.9–1.1 was considered as additive, a ratio <0.9 as antagonistic and a ratio >1.1 as synergistic (Golla et al., 2021).

## Results

### ONC201/TIC10 Inhibits the Cellular Viability of Medulloblastoma Cells Independent of C-Myc Expression

**FIGURE 1:**
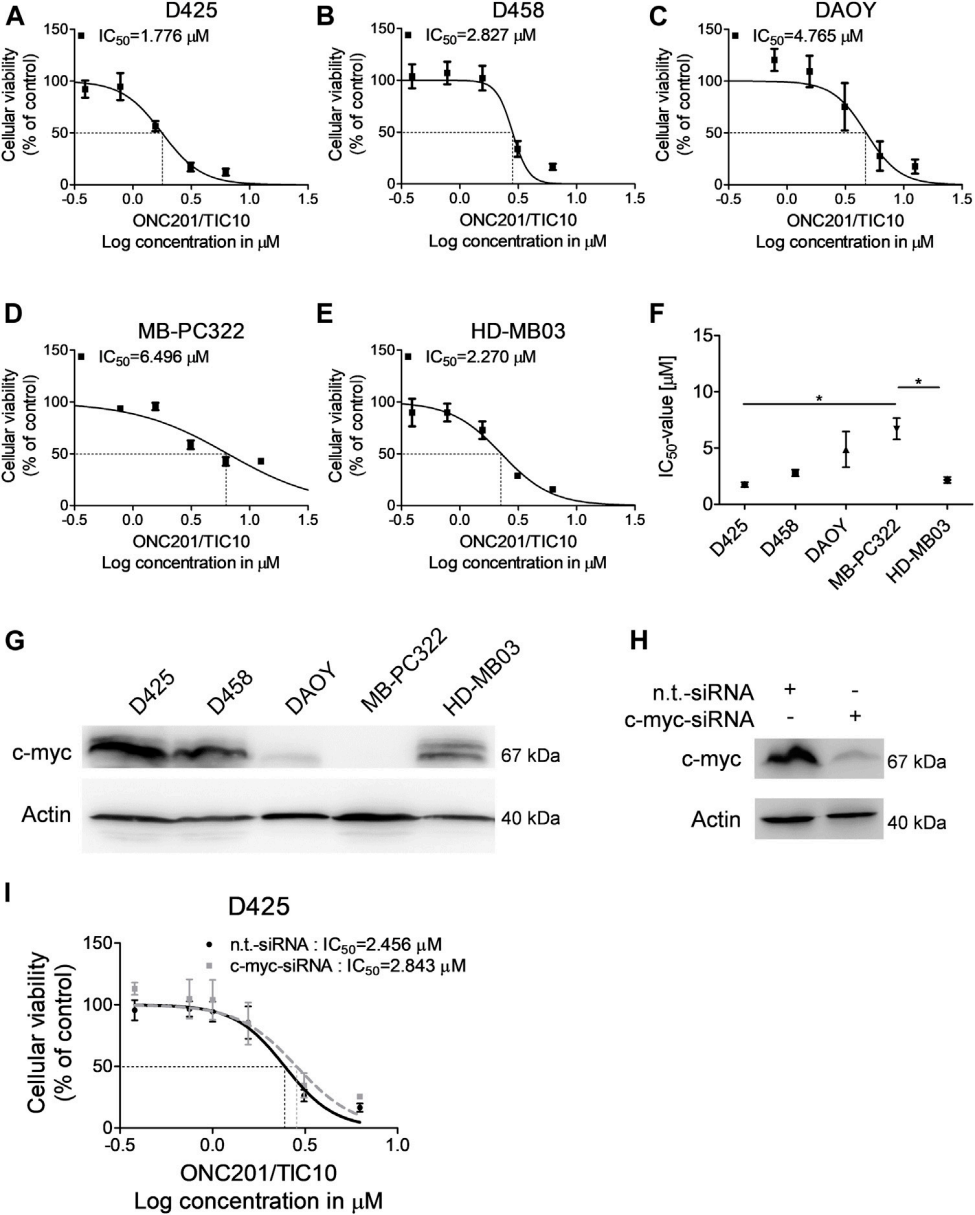
ONC201/TIC10 has a dose-dependent anti-proliferative effect on medulloblastoma cells. **(A–E)**, D425, D458, DAOY, MB-PC322 and HD-MB03 cells were treated with solvent or increasing concentrations of ONC201/TIC10 under serum starvation (1.5%FBS). Non-linear regression was performed and IC_50_-values were calculated. Data are presented as mean and SEM of three independent experiments. **(F)**, Quantitative representation of the IC_50_-values for indicated cells treated as described for **(A–E)**. Mean, SEM and *n* = 3. *p < 0.05. **(G)**, Whole cell extracts of D425, D458, DAOY, MD-PC322 and HD-MB03 cells were collected and subjected to Western blot analysis for c-myc and Actin. **(H)**, D425 cells were transfected with non-targeting (n.t.)-siRNA or c-myc-siRNA. Cell lysates were collected and Western blot analysis for c-myc and Actin was performed. **(I)**, D425 cells were treated as described for H prior to treatment with solvent or increasing concentrations of ONC201/TIC10. Non-linear regression was performed and IC_50_-values were calculated. Data are presented as mean and SD of three independent experiments.

Imipridones such as ONC201/TIC10 have been shown before to impair the cellular viability of different malignancies including glioblastoma, colorectal cancer, or ovarian cancer. In glioblastoma, a direct correlation was found between the response towards ONC201/TIC10 and c-myc expression (Ishida et al., 2018). Notably, upregulation of myc represents a molecular feature that has been shown to be associated with a worse outcome in medulloblastoma (Cavalli et al., 2017; Northcott et al., 2019). We therefore sought to examine whether ONC201/TIC10 has the ability to inhibit the cellular viability of medulloblastoma cells expressing varying levels of c-myc. D425, metastatic D458, DAOY, and HD-MB03 medulloblastoma cell lines as well as MB-PC322 primary cultured medulloblastoma cells were treated with increasing concentrations of ONC201/TIC10 prior to performing MTT assays (Figures 1A–E). As shown in Figures 1A–E, a sigmoid dose response was noted in all cells with IC_50_-values ranging from approximately 1.8–6.5 µM. Notably, c-myc expression was high in D425, D458, and HD-MB03 cells but low in DAOY and MB-PC322 cells (Figure 1G). While the IC_50_-value of ONC201/TIC10 in MB-PC322 cells was statisticallysignificant different from D425 and HD-MB03 cells no significant difference regarding the response towards ONC201/TIC10 was found when comparing the IC_50_-value for DAOY cells with the IC_50_-values for the other cells (Figure 1F).

Therefore, in order to further assess whether c-myc expression affects the response of medulloblastoma cells towards ONC201/TIC10, specific knock down of c-myc was performed in D425 cells (Figure 1H). As shown in Figure 1I, the IC_50_-value for ONC201/TIC10 in D425 cells that were silenced for c-myc was not markedly different when compared to cells treated with non-targeting siRNA.

### ONC201/TIC10 Suppresses OXPHOS in Medulloblastoma Cells

**FIGURE 2:**
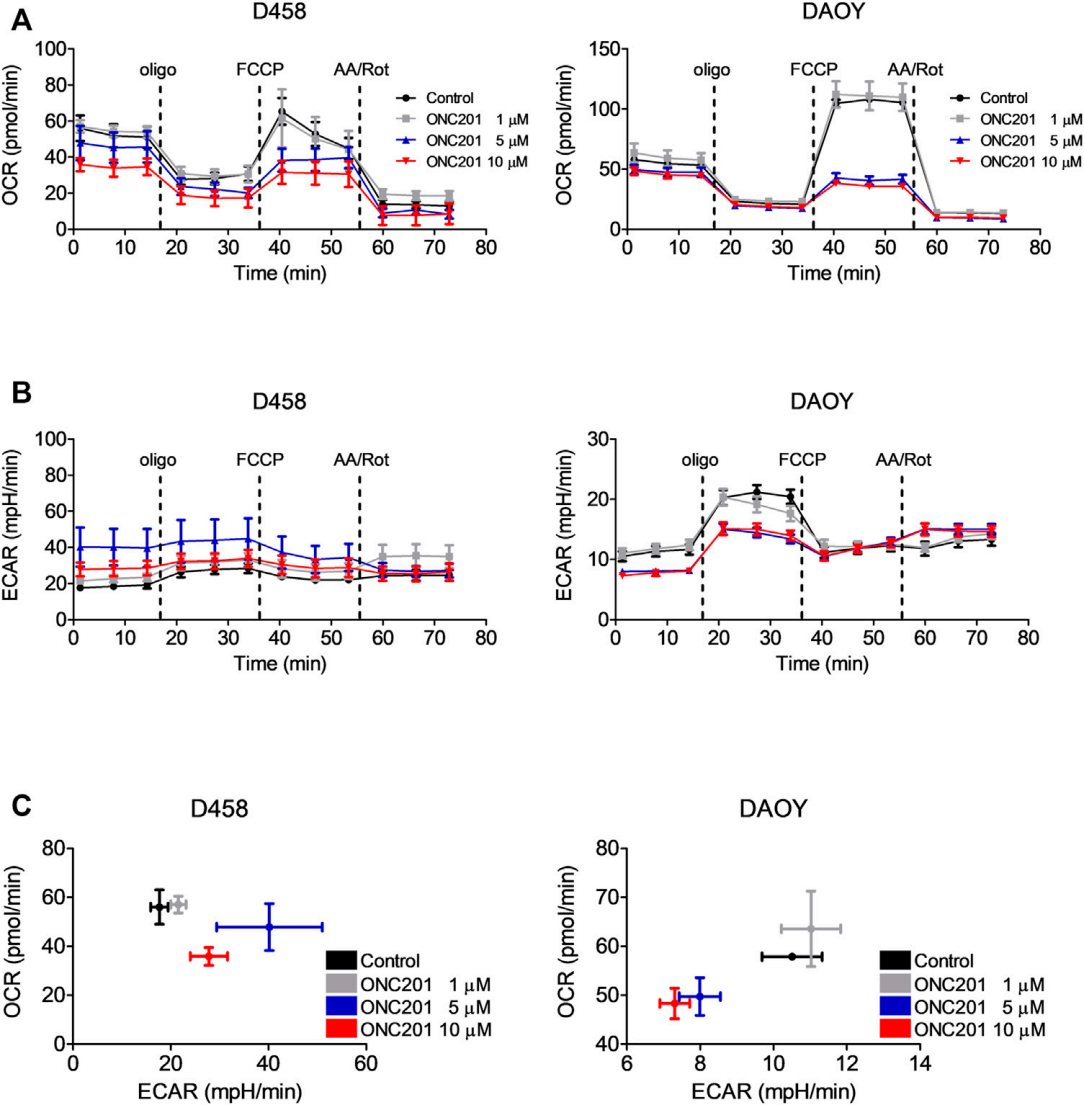
ONC201/TIC10 suppresses OXPHOS and differentially modulates the glycolytic rates in D458 an DAOY cells. **(A)**, D458 and DAOY cells were treated for 24 h with indicated concentrations of ONC201/TIC10. Mitochondrial stress tests were performed. Oxygen consumption rates (OCR) were continuously recorded while oligomycin (olig), FCCP and antimycin A/rotenone (AA/Rot) were sequentially injected into the wells. Mean and SD of four technical replicates representative for two independent experiments. **(B)**, Extracellular acidification rates (ECAR) for cells treated as described for A. Mean and SD of four technical replicates representative for two independent experiments. **(C)**, D458 and DAOY cells were treated for 24 h with indicated concentrations of ONC201/TIC10. Graphical representation of baseline OCR/ECAR-values. Mean and SD of four technical replicates representative for two independent experiments.

In other malignancies, the anti-cancer activity of ONC201/TIC10 has been linked to an impairment of oxidative phosphorylation and ATP depletion resulting from a hyperactivation of the mitochondrial protease CIpP and an increased depletion of respiratory chain complexes. To address the question whether ONC201/TIC10 affects oxidative phosphorylation in medulloblastoma cells, we performed extracellular flux analyses in D458 and DAOY cells. As shown in Figure 2A, treatment with increasing concentrations of ONC201/TIC10 results in a marked decrease in oxidative consumption rates in both cell lines at baseline and throughout the mitochondrial stress test.

### ONC201/TIC10 Reduces the Expression of Complexes of the Respiratory Chain in Medulloblastoma Cells

**FIGURE 3:**
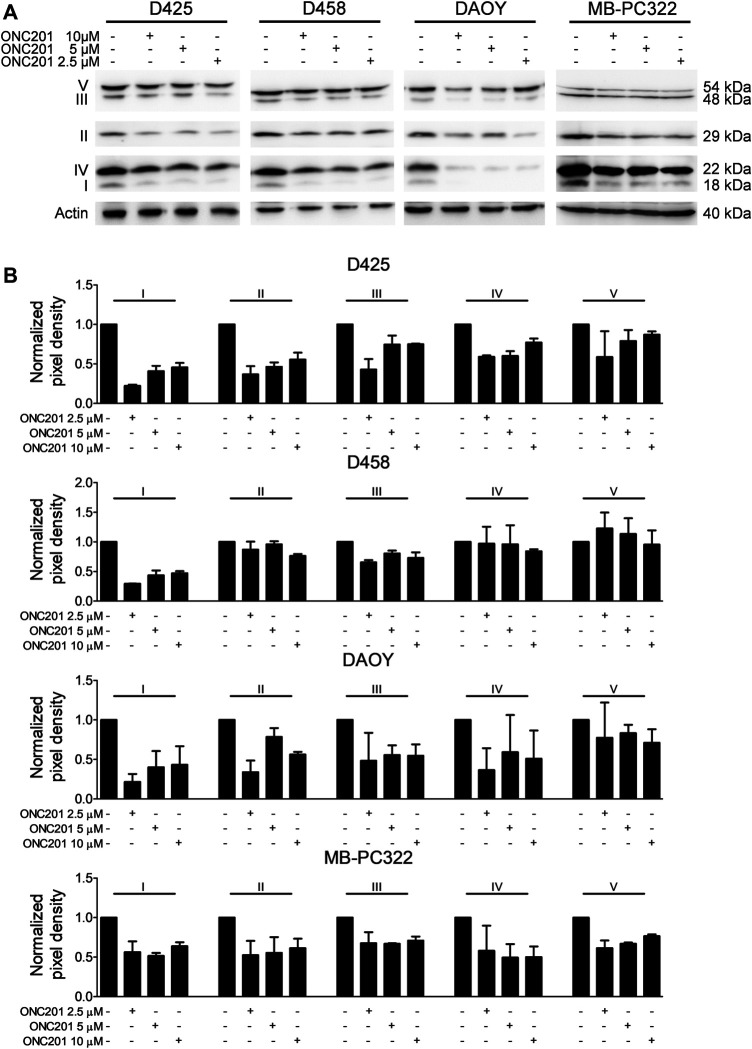
ONC201/TIC10 reduces the expression of respiratory chain complexes. **(A)**, D425, D458, DAOY and MB-PC322 cells were treated for 24 h as indicated. Cell lysates were collected and analysed by Western blot for the expression of respiratory chain complexes I-V. Equal loading was confirmed by analysis for Actin expression. **(B)**, Quantitative representation of cells treated as described for A. Densitometric analysis was performed using ImageJ (NIH, Bethesda, MD; http://imagej.nih.gov/ij). Data were normalized to control. Columns: mean. Error bars: SD. *N* = 2.

A reduction in oxidative phosphorylation following treatment with ONC201/TIC10 has been shown in other models to go along with changes in the expression of respiratory chain proteins. To verify, whether this proposed part of the mechanism of action of ONC201/TIC10 also holds true for this setting, Western blot analysis for respiratory enzymes was performed. Treatment with increasing concentrations of ONC201/TIC10 resulted in a reduced expression of various respiratory chain complexes but did not follow a dose response under these conditions (Figures 3A,B). The most consistent observation was an ONC201/TIC10-mediated reduced expression of complexes I and III (Figure 3B). While treatment with ONC201/TIC10 decreased the expression of complexes II and IV in D425 and DAOY cells, it was not altered in metastatic D458 cells (Figure 3B). Complex V (ATP synthase) levels were only slightly reduced in D425 and DAOY cells when treated with ONC201/TIC10. In MB-PC322 cells, treatment with ONC201/TIC10 led to a reduced expression of all complexes of the respiratory chain.

### ONC201/TIC10 Differentially Affects the Basic Glycolytic Rate in Medulloblastoma Cells

We next analysed the effects of ONC201/TIC10 on the glycolytic rates of D458 and DAOY medulloblastoma cells using the extracellular acidification rates (ECAR) as a surrogate. While treatment with ONC201/TIC10 induced a decrease in OXPHOS in both cell lines, the glycolytic rates were inversely altered by this drug (Figures 2B,C). At base-line, ECAR was upregulated following treatment with ONC201/TIC10 in D458 cells. In contrast, in DAOY a decrease of the ECAR was observed.

### Glucose Deprivation Sensitizes Medulloblastoma Cells Towards ONC201/TIC10

**FIGURE 4:**
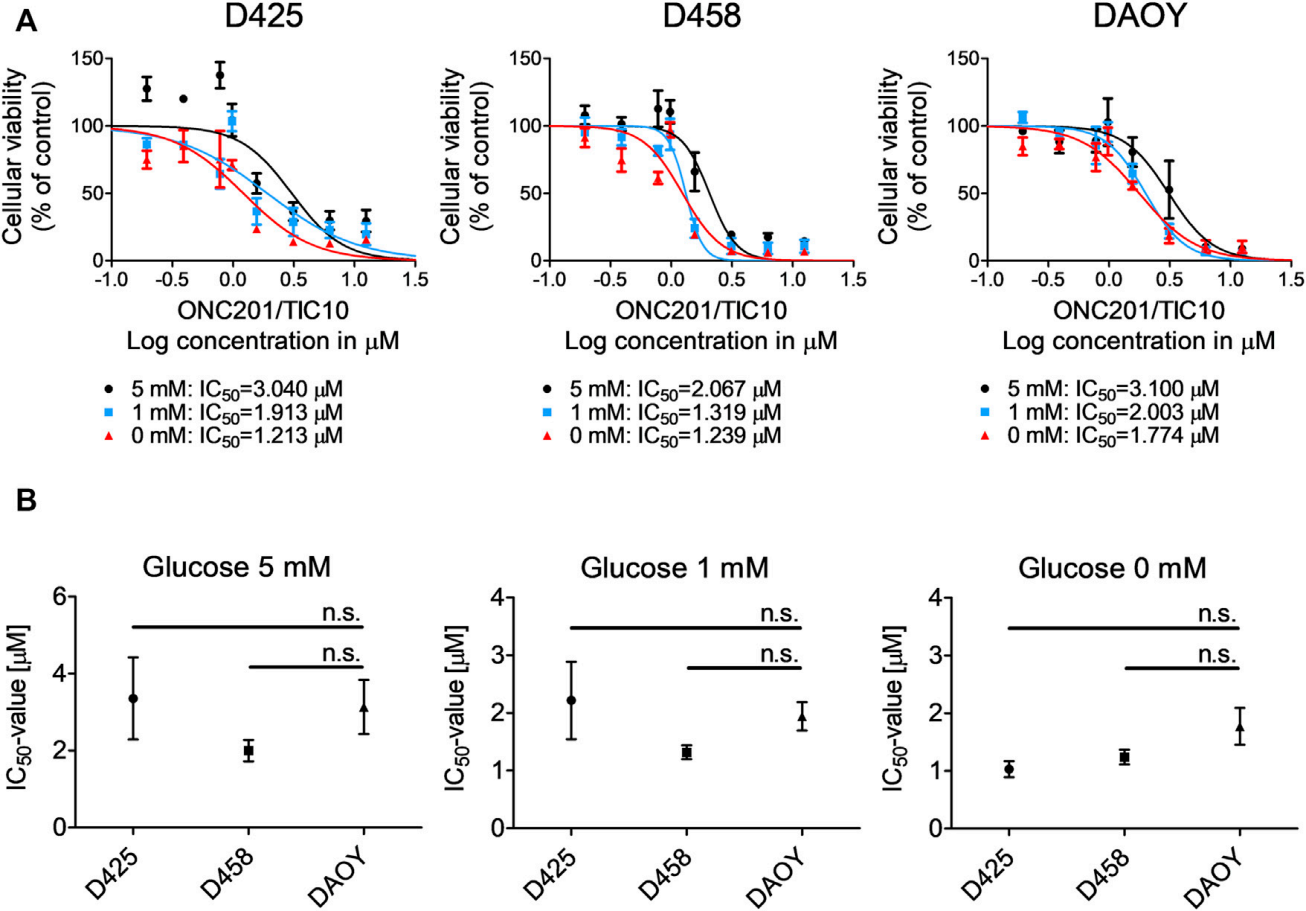
Glucose starvation sensitizes for ONC201/TIC10. **(A)**, D425, D458 and DAOY cells were treated with solvent or increasing concentrations of ONC201/TIC10 under serum starvation (1.5%FBS) and indicated glucose concentrations. Non-linear regression was performed and IC_50_-values were calculated. Data are presented as mean and SEM of three independent experiments. **(B)**, Quantitative representation of the IC_50_-values for indicated cells treated as described for A. Mean, SEM and *n* = 3.

Given our observation that ONC201/TIC10 modifies core metabolic pathways in different medulloblastoma cells, we formed the hypothesis that this compound creates a metabolically vulnerable cellular state which may be further destabilized by deprivation of glucose as a major energy substrate. To test this hypothesis, we exposed medulloblastoma cells to increasing concentrations of ONC201/TIC10 in the presence of decreasing levels of glucose (Figure 4A). Our data show that the lower the glucose concentrations are, the more the dose response curves are shifted to the left side and the more the IC_50_-values decrease. Notably, the IC_50_-values did not significantly vary among the three cell lines tested even under glucose-starved conditions despite varying expression of c-myc (Figure 4B).

### ONC201/TIC10 Combined With 2-Deoxyglucose Synergistically Impairs the Cell Viability of Medulloblastoma Cells

**FIGURE 5:**
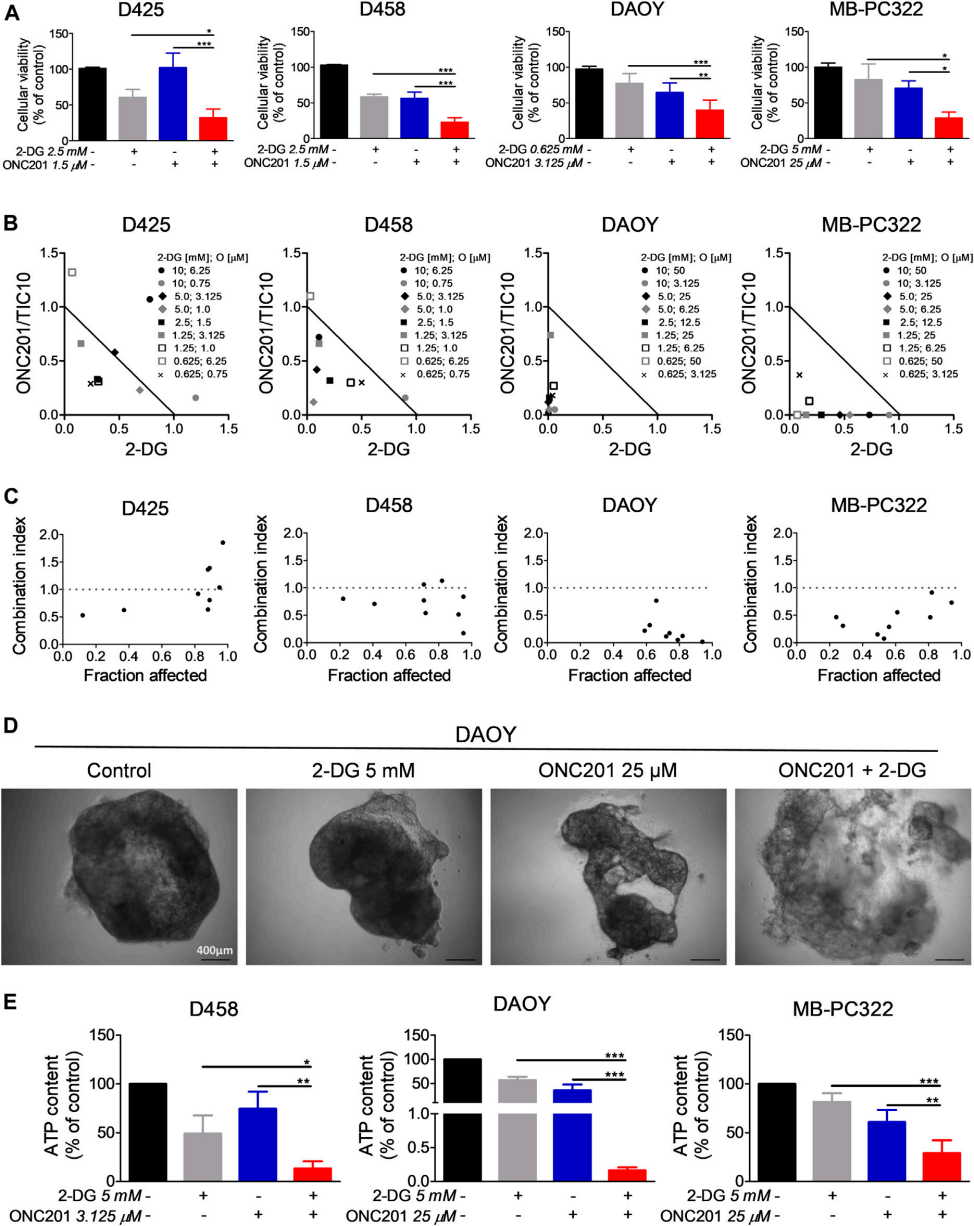
2-Deoxyglucose and ONC201/TC10 synergistically inhibit the cellular viability and the growth of spheroids in medulloblastoma. **(A)**, D425, D458, DAOY and MB-PC322 cells were treated with solvent or indicated concentrations of 2-Deoxyglucose and/or ONC201/TIC10 under serum starvation (1.5%FBS) for 72 h prior to performing MTT assays. Columns: mean. Error bars: SD. *N* = 3. *p < 0.05, **p < 0.01, ***p < 0.005. **(B)**, D425, D458, DAOY and MB-PC322 cells were treated for 72 h as indicated. MTT assays were performed and normalized isobolograms were calculated. Data points within the triangle are indicative for a synergistic, data points outside of the triangle point towards an antagonistic and data points on the diagonal line indicate an additive drug-drug interaction. Data are representative for three independent experiments. **(C)**, Combination index (CI)-plots for medulloblastoma cells treated as described for B. A CI < 1 signifies a synergistic, a CI = 1 an additive and a CI > 1 an antagonistic drug-drug interaction. **(D)**, Microphotographs of DAOY spheroids that were grown for 14 days. Treatment as indicated was performed on d 7, d 9, and d 12. Magnification, × 4. **(E)**, D458, DAOY and MB-PC322 spheroids were treated as described for D. CellTiter-Glo^®^ assays were performed to determine the ATP content. Columns: mean. Error bars: SD. *N* = 3. *p < 0.05, **p < 0.01, ***p < 0.005.

Based on our observations that 1) glucose deprivation sensitizes for ONC201/TIC10 and 2) ONC201/TIC10 upregulates the glycolytic rate in D458 cells, we decided to assess whether the anti-neoplastic activity of ONC201/TIC10 can be significantly enhanced by a concomitant treatment with the glycolysis inhibitor 2-Deoxyglucose. To this end, we performed cell viability assays in medulloblastoma cells treated with ONC201/TIC10 and 2-Deoxyglucose alone or combined. As shown in Figure 5A, D425, D458, DAOY and MB-PC322 cells treated with the combination of ONC201/TIC10 and 2-Deoxyglucose displayed a statistically significant reduction of the cell viability when compared to cells receiving treatment with the single agents or solvent. Moreover, isobolograms and combination index-plots were calculated and revealed that ONC201/TIC10 and 2-Deoxyglucose act predominantly in a synergistic manner with respect to their anti-medulloblastoma activity (Figures 5B,C).

### ONC201/TIC10 Combined With 2-Deoxyglucose Synergistically Inhibits the Growth of Spheroids

Next, we examined how the combination treatment with ONC201/TIC10 and 2-Deoxyglucose affects the growth of medulloblastoma cells in a 3-dimensional setting. For this purpose, spheroids were established prior to treatment with the two drugs. Microscopic imaging showed that spheroids treated with the combination developed a reduced cellular density and experienced a disruption of the spheroid structure (Figure 5D). In line with this finding, the ATP content of D458, DAOY and MB-PC322 spheroids treated with the combination therapy was significantly reduced in comparison with the single agent treatments and control (Figure 5E). Furthermore, BLISS analysis revealed that ONC201/TIC10 and 2-Deoxyglucose did act in a synergistic manner in spheroids derived from D458 (BLISS index = 1.37), DAOY (BLISS index = 1.26) and MB-PC322 (BLISS index = 1.41) cells.

## Discussion

Medulloblastoma represents one of the most common childhood brain tumors and while we have learned a lot regarding the molecular characteristics of this disease within the past years more efficient and less toxic therapeutics are greatly needed (Northcott et al., 2019; Orr, 2020). Patients with medulloblastomas harboring amplified myc were reported to face a worse prognosis (Cavalli et al., 2017). It was on these grounds, that we formed the hypothesis that the imipridone ONC201/TIC10, which was shown before to be more potent in c-myc high expressing glioblastomas (Ishida et al., 2018), might have a pronounced anti-medulloblastoma efficacy in this subtype.

Our data showed that ONC201/TIC10 had strong anti-neoplastic activity across multiple medulloblastoma cell models. However, we did not find a statistically significant difference between c-myc low-expressing DAOY cells and c-myc high-expressing D425, D458, and HD-MB03 cells. In addition, silencing of c-myc in D425 cells did not significantly alter the response towards ONC201/TIC10. These data suggest that c-myc expression does not predict the response towards ONC201/TIC10 in this setting. In contrast to our findings, Ishida et al. reported that c-myc high-expressing glioblastoma cells were significantly more responsive towards a treatment with ONC201/TIC10 (Ishida et al., 2018). Moreover, silencing of c-myc using shRNA markedly increased DNA fragmentation following ONC201/TIC10 treatment. The discrepancy between the results of this study and the study by Ishida et al. are likely due to differences in the genetic background of these two different tumor entities. Taken together, it also suggests that c-myc might not be as important with respect to the mechanism that underlies the activity of ONC201/TIC10 in medulloblastoma when compared to glioblastoma.

Another controversy, is represented by the fact that while OXPHOS seems to be consistently suppressed by ONC201/TIC10, ECARs as an outread for the glycolytic rate show a varying response. Treatment of U87 and SF188 glioblastoma cells with ONC201/TIC10 was reported to result in significantly decreased ECARs (Ishida et al., 2018). In contrast, in U251 and A172 glioblastoma cells ECARs at baseline were markedly increased when cells were subjected to ONC201/TIC10 (Pruss et al., 2020). In all these cells, OXPHOS was consistently shown to be suppressed by ONC201/TIC10 independent of differences in experimental conditions. Similarly, in the present study ONC201/TIC10 led to a strong down-regulation of OXPHOS in all medulloblastoma cell lines tested. However, while ONC201/TIC10 up-regulated ECAR in D458 cells, a decrease was observed in DAOY cells. The origin of this discrepancy is presently not identified. Differences in experimental conditions, genetic or metabolic background are likely to be involved and need further elucidation.

We have shown before that 2-Deoxyglucose synergistically enhanced the inhibitory activity of ONC201/TIC10 on the cellular viability and tumor growth in glioblastoma (Pruss et al., 2020). These biological effects are in line with the observations we made in the current study. Interestingly, in this setting, a differing response regarding glycolytic rates following treatment with ONC201/TIC10 did not seem to translate into markedly different responses towards an additional treatment with the glycolysis inhibitor 2-Deoxyglucose. Regardless whether ONC201/TIC10 led to an increase of the ECAR as seen in D458 or to a decrease of the ECAR as seen in DAOY, a predominantly synergistic anti-neoplastic activity of the combination was observed. As a consequence, the hypothesis that the mechanistic basis of the synergistic effect of the combination therapy relies only on an additional inhibition of glycolysis needs to be at least questioned and indicates that other molecular or metabolic alterations might be involved. Further analyses of the transcriptome and the metabolome of D458 and DAOY cells subjected to the combination therapy are planned and will likely aid at uncovering additional modes of mechanism.

Our data provide evidence that interference with OXPHOS and glycolysis might represent a useful instrument for the treatment of medulloblastoma patients. In order to guide therapeutic measures, novel technologies are in development to facilitate a metabolic fingerprinting. As an example, varying substrates were developed such as ferric or magnetic particles covered with a Pd-Au shell to improve the analysis of metabolites in body fluids by laser desorption/ionization mass spectrometry (Cao et al., 2020; Huang et al., 2020; Pei et al., 2020). These analyses combined with machine learning tools allowed for the identification of specific metabolic patterns in patients with early stage lung adenocarcinoma (Huang et al., 2020), medulloblastoma (Cao et al., 2020) and gynecological cancers (Pei et al., 2020). In the future, this technology may allow for a high-throughput and cost-efficient way of identifying patients with tumors showing specific metabolic signatures that may predict a response towards drugs targeting the tumor cell metabolism such as ONC201/TIC10 or 2-Deoxyglucose. In addition, changes in metabolic signatures following therapy could be monitored and used to adapt the therapeutic interventions in a dynamic fashion.

The development of therapeutic resistance represents a major problem for the treatment of malignant diseases such as medulloblastoma and will very likely also extend to anti-metabolic approaches. Therefore, preventive strategies should be considered. For instance the use of nanodrugs or Janus particles have been described to allow for a better drug delivery and drug distribution across tumor tissue to avoid suboptimal local drug dosing and a subsequent selection of resistant clones (Su et al., 2019; Ma et al., 2020). In addition, the combination of anti-metabolic strategies with treatment modalities such as photodynamic or sonodynamic therapy using a different anti-neoplastic mechanism may add further therapeutic benefits and counteract therapeutic resistance (Wu et al., 2020).

There are certainly a number of limitations that need to be carefully considered when interpreting the results of this study. One limitation is presented by the fact that we included only five different medulloblastoma cells in our analyses. Moreover, we did not verify our findings in an *in vivo* model. However, to our knowledge, this study provides first evidence for ONC201/TIC10 as a potentially valuable therapeutic in medulloblastoma. In addition, proof of principle is provided for a multi-targeted anti-metabolic therapeutic strategy resulting in a synergistic anti-neoplastic activity in this setting. This approach is supported by the fact that both, ONC201/TIC10 and 2-Deoxyglucose are known to cross the blood brain barrier reaching concentrations that are in the same range as the ones used in this study. Moreover, both drugs were used in clinical trials with a good safety profile (Singh et al., 2005; Raez et al., 2013; Arrillaga-Romany et al., 2017; Arrillaga et al., 2018; Chi et al., 2019). Of course, a combined treatment with multiple drugs that target core metabolic pathways holds the risk for unforeseen toxicity and needs further critical validation. However, the results of this study suggest that translation of ONC201/TIC10 into the clinics for the treatment of patients with medulloblastoma—independent of myc status—might hold promise and legitimate further studies.

## Data Availability

The raw data supporting the conclusion of this article will be made available by the authors, without undue reservation.
